# Hearing impairment following radiotherapy in patients with nasopharyngeal carcinoma (Review)

**DOI:** 10.3892/mco.2026.2970

**Published:** 2026-08-11

**Authors:** Lixiang Liu, Yao Ke, Jin Du, Yili Li, Yahua Zhong, Lei Zhang

**Affiliations:** Hubei Key Laboratory of Tumor Biological Behaviors, Department of Head, Neck and Pediatric Oncology, Hubei Cancer Clinical Study Center, Zhongnan Hospital of Wuhan University, Wuhan, Hubei 430071, P.R. China

**Keywords:** nasopharyngeal carcinoma, radiotherapy, hearing loss

## Abstract

Nasopharyngeal carcinoma (NPC) is a geographically prevalent malignant mucosal tumor endemic to southern China and Southeast Asia. Radiotherapy constitutes the core curative treatment for NPC, often combined with chemotherapy or targeted therapy in clinical practice. Hearing loss is a common radiotherapy-related complication caused by radiation damage to middle ear structures, inner ear cells and auditory nerves, with a variable incidence affected by treatment protocols and patient characteristics. Such auditory dysfunction negatively impacts patients' cognition, emotion and quality of life. However, systematic summaries of subjective evaluation tools and targeted non-surgical rehabilitation strategies for radiotherapy-induced hearing impairment remain insufficient. Accordingly, this review aims to elaborate on the classification, multidimensional clinical impacts, subjective assessment scales, and non-surgical interventions of radiotherapy-related hearing loss, so as to provide evidence-based references for clinical rehabilitation of NPC survivors.

## 1. Introduction

Compared with other types of malignancies, nasopharyngeal carcinoma (NPC) occurs infrequently and is ranked as the 22nd most prevalent cancer worldwide (1,2). However, the incidence rate of NPC ranks first among different types of head and neck cancer (3). According to statistics from the International Agency for Research on Cancer, there were ~129,000 new cases of NPC in 2018, with >70% of the new cases occurring in East Asia and Southeast Asia, indicating a geographical imbalance (4). Based on the burden and trend of NPC in China from 1990 to 2019, the crude incidence rate has increased from 2.71 per 100,000 to 7.76 per 100,000 individuals, highlighting an increasing trend (5). The disease imposes a substantial global mortality burden, with ~80,000 annual NPC-related mortalities worldwide (6). China, as the most endemic country, accounts for >43% of global NPC mortalities, with 28,000 to 34,800 annual mortalities and a national age-standardized mortality rate of 1.18-1.5 per 100,000 population (7). Radiation therapy is considered the primary treatment modality for NPC, demonstrating an overall survival rate of up to 85% after 5 years for patients with early-stage disease (8). However, patients often experience long-term side effects following radiation therapy. For example, hearing impairment is the second most common long-term side effect after xerostomia (9). Hearing impairment impacts the cognitive function and emotional well-being of patients and diminishes their quality of life. Therefore, the present study aimed to comprehensively review the classification of hearing impairment following radiotherapy for NPC and investigated the assessment tools and intervention measures used in practice. The present review may provide guidance for clinical practice and aid in the rehabilitation of patients following radiotherapy.

## 2. Categories of hearing loss

Hearing loss is classified into three types based on pathological mechanisms, namely: i) Conductive hearing loss (CHL); ii) sensorineural hearing loss (SNHL); and iii) mixed hearing loss (MHL) (10). Healthy hearing is considered when both air conduction and bone conduction thresholds are <25 decibels (dB). An increased air conduction threshold, a bone conduction threshold at the expected level and an air-bone gap >10 dB are indicative of CHL. SNHL is considered when both the air and bone conduction thresholds are increased, but the difference between them is <10 dB. An increased air and bone conduction threshold accompanied by an air-bone gap of >10 dB indicates MHL. Among these, radiation therapy may result in patients developing CHL, while a combination of chemotherapy and radiotherapy may result in MHL (11,12).

### CHL

During radiotherapy for NPC, the primary radiation target encompasses the middle ear structures, including the tympanic cavity, Eustachian tube, mastoid sinus and petrous apex (13). The middle ear serves a pivotal role in sound transmission by converting sound waves from the outer ear into mechanical waves for further conduction. Radioactive damage to the middle ear may lead to CHL, and radiation-induced otitis media is considered the predominant clinical manifestation (14). Previous studies demonstrate that radiation-induced injury primarily affects the Eustachian tube and tympanic cavity, where direct radiation damages both mucosa and cartilage of the Eustachian tube (15,16). In addition, radiation damages the innervating muscles, including the tensor veli palatini muscle, resulting in an impaired opening function of the Eustachian tube (17). Radiation injury to the middle ear mucosa, blood vessels and lymphatic endothelium disrupts the local mucociliary clearance system. This process not only increases tissue fluid exudation, but also slows lymphatic drainage, eventually leading to retention and obstruction of secretions. Radiation-induced necrosis of the nasal-pharyngeal or sinus mucosa may result in a bacterial infection (18), which subsequently involves the Eustachian tubes (19). The interplay between the aforementioned pathological processes leads to secretory otitis media, resulting in CHL. Post-radiotherapy, CHL is typically transient, with recovery of Eustachian tube function and improvement in middle ear effusion. As the negative pressure within the middle ear is relieved, hearing loss resolves. The development of atrophic (fibrotic) otitis media and ossicular chain necrosis after radiotherapy is multifactorial, which explains why these complications only arise in partial patients rather than all cases. Based on clinical cohort studies focusing on head and neck radiotherapy, the overall incidence of radiation-related atrophic/fibrotic otitis media is approximately 1 out of 5 to 1 out of 4 patients (20).

### SNHL

SNHL is caused by damage to the cochlea and auditory nerve (21). Following radiotherapy, SNHL is categorized into two types based on its time of onset, namely: i) Early-onset (acute); and ii) late-onset (chronic). Early-onset SNHL may occur within hours to 1 week after exposure to ionizing radiation and, in certain cases, this type of hearing loss may be reversible or partially reversible. Conversely, late-onset SNHL manifests as a chronic, progressive and irreversible process that arises within 6-24 months post-irradiation (22,23). In a number of patients, complete deafness may develop within weeks or months (24). The reported incidence rate of post-radiotherapy SNHL varies between 15 and 30%, with a notably increased occurrence rate observed in patients receiving cisplatin-based chemotherapy compared with those receiving radiotherapy alone (25,26). Additionally, >50% of patients that suffer with SNHL will experience permanent SNHL (27). SNHL develops through complex mechanisms that are not fully understood. Possible contributing factors include blood vessel abnormalities, atrophy of the spiral ligament and basilar membrane, loss of outer hair cells and deterioration of the eighth cranial nerve (28).

### MHL

MHL is a composite manifestation of both CHL and SNHL, which typically occurs at the same time as damage to the outer or middle ear. MHL impacts the cochlea and auditory nerve, further complicating the intricacy and challenges associated with treating auditory impairment (29).

## 3. Implications of hearing loss

### Impact of hearing loss on the quality of life of patients

Hearing loss can impair the communication abilities of patients, leading to limited participation in everyday listening situations. This may affect daily functions and negatively impact the quality of life of the patient (30). A study by Sano *et al* (31) reveals the limitations of social and daily activity experienced by individuals with hearing loss compared with those without hearing loss, from both a physiological and psychological perspective (31). In addition, a study by Wie *et al* (32) reports that severe unilateral hearing impairment, such as deafness, notably disrupts speech perception, communication skills and social interactions among patients with hearing loss compared with those without hearing loss (32). In addition, symptoms such as dizziness and tinnitus, which are associated with sudden SNHL (SSHL), may also contribute to anxiety surrounding the potential recurrence or fear of hearing loss in the unaffected ear, further impacting the quality of life of patients (33).

### Potential association between hearing loss and mental disorders

A number of studies substantiate the association between hearing loss and neurocognitive impairments (34,35). Compared with patients without hearing loss, individuals with moderate to severe hearing loss who do not utilize hearing aids exhibit impaired language expression, increased social isolation and accelerated cognitive decline (36). A multi-center, parallel-group, randomized controlled trial conducted in the United States investigated untreated hearing loss among adults aged 70-84 years without severe cognitive impairments. The findings demonstrate that interventions, such as provision of hearing aids and health education, may mitigate the risk of cognitive decline within a 3-year period, promoting stability in the cognitive capacities of older patients (37). A further study examining the association between hearing aid usage and cognitive abilities reveals that patients who wear hearing aids exhibit a notably reduced risk of cognitive decline, compared with those lacking auditory correction during a long-term follow-up period ranging from 2-25 years (38). This long-term protective association has been validated by recent population-based longitudinal cohorts and clinical observational research (38). Large-sample prospective evidence verified that standardized hearing aid intervention effectively cut down dementia and global cognitive deterioration risk in elderly hearing-impaired individuals during multiyear follow-up (39). Additionally, real-world cohort data focusing on middle-aged and elderly subjects further confirmed sustained hearing aid application independently alleviates progressive cognitive impairment and brain function regression in long-term observational trials (40). Moreover, an analysis elucidating the potential association between auditory restoration and changes in short-term cognitive test scores indicates a notable 3% improvement following hearing aid use (24), further corroborating the positive impact of auditory intervention on cognitive function.

Previous studies reveal an association between anxiety and hearing loss, with generalized anxiety being particularly prominent (41,42). Compared with individuals without hearing loss, those with mild hearing impairment may exhibit a markedly increased incidence of anxiety, which may also increase as the degree of hearing loss increases (41). Hearing loss weakens cognitive reserves, exacerbates executive dysfunction and disrupts normal emotional responses and regulatory mechanisms, leading to decreased cognitive performance and increased risk of depression (42).

A 5-year follow-up study demonstrated that patients with existing or deteriorating hearing loss exhibited far more noticeable progression of depressive symptoms than those with intact hearing (43). Meanwhile, aggravated depressive symptoms were accompanied by increased hearing loss at low and mid frequencies, implying a potential correlation between the two conditions (44). A previous study, focusing on the mental health of adolescents, aged 10 to 19 years, also reveals an association between mental health and auditory function (45). The survey conducted among adolescent patients with impaired auditory function reveals that 30% of patients exhibit symptoms of depression, such as persistent low mood, social withdrawal, poor concentration and loss of interest in daily activities, while 21% of patients have symptoms of anxiety, including excessive worry, restlessness and palpitations. Moreover, older adolescents (aged 15 to 19 years) score higher on measures for depressive symptoms than younger adolescents with hearing loss. Among the group with severe to profound auditory impairments, the incidence rates for both depression and anxiety were more pronounced (45). Additionally, results of a previous study reveal that the prevalence rate for depression in deaf or hard-of-hearing populations is ~7%, whereas the lifetime prevalence of depression in this group can be as high as 26% (46). Children who experience ridicule due to their impaired auditory function or other disabilities, such as visual impairments, neurological disorders or developmental delays during upbringing, may face an increased risk of developing depression compared with those who do not endure such ridicule (46).

## 4. Objective evaluation tools for hearing loss

### Hearing handicap inventory for adults screen (HHIA-S)

The HHIA-S is a preliminary diagnostic scale for hearing impairment, which is used worldwide (47). This scale comprises 10 items, including five emotional and five social/contextual aspects (Table I). The scoring criteria are as follows: A response of ‘yes’ receives four points; a response of ‘sometimes’ receives two points; and a response of ‘no’ receives zero points. The total score ranges from 0-40, with higher scores indicating more severe hearing loss. A score of more or equal to eight suggests the presence of an impairment, while scores between eight and 24 indicate mild impairment, and scores >24 indicate severe impairment (47). In China, the HHIA-S was translated in 2014(48), a score >8 on the scale was used as the cut-off to identify participants with hearing loss, those scoring exactly 8 were classified as having normal hearing. When conducting hearing screens for individuals with an average pure tone air conduction threshold (pure tone average; PTA) of >40 dB Hertz, HHIA-S is a user-friendly self-assessment questionnaire that can be administered without the need for specialized personnel or equipment, thereby conserving medical resources (47). However, this method may exhibit certain limitations, as subjective evaluations may introduce inherent biases, and specific types of hearing impairments including conductive hearing loss, retrocochlear hearing impairment, mixed hearing loss, subclinical noise-induced or ototoxic sensorineural hearing loss, asymmetric hearing loss, auditory processing disorder, as well as hearing dysfunction in children and cognitively impaired populations still require integration with objective screening methods to achieve accurate identification and classification (49).

### Seven-item Eustachian tube dysfunction questionnaire (ETDQ-7)

The ETDQ-7 is a valuable tool for evaluating symptoms associated with abnormal Eustachian tube function, encompassing issues such as ear fullness, ear pain, autophony and hearing loss (50). This comprehensive survey comprises seven questions that gauge the severity of these subjective symptoms on a scale ranging from one to seven, where higher scores indicate more pronounced manifestations (Table II). A study by Cao *et al* (51) utilized the Chinese version of the ETDQ-7 to assess patients with Eustachian tube dysfunction alongside healthy individuals. The results of the aforementioned study demonstrates that the Chinese version of the ETDQ-7 exhibits high levels of reliability (coefficient, 0.879), sensitivity (96.1%) and specificity (85%). Therefore, these results indicate the efficacy of utilizing the Chinese version of the ETDQ-7 in evaluating Eustachian tube dysfunction.

The ETDQ-7 may be used to gather information on the symptoms experienced by patients in an efficient manner, with comprehensible content and convenient completion. However, relying only on the ETDQ-7 scores does not allow for a differentiation between obstructive and patulous Eustachian tube disorders. This limits its utility as a diagnostic tool for obstructive Eustachian tube dysfunction or an objective measurement method for Eustachian tube function (52).

### Patient-reported outcomes version of the common terminology criteria for adverse events (PRO-CTCAE)

Ototoxicity constitutes a treatment-related adverse event induced by antitumor therapies. The ototoxicity grading criteria assess patients' symptomatic conditions in the week prior to each clinical assessment, incorporating three evaluation dimensions: Symptom frequency, severity and interference with daily functioning. This grading system adopts a 1-5 point rating scale (Table III) (53). A higher score indicates more pronounced symptoms. There are multiple versions of the PRO-CTCAE assessment, which also has extensive dissemination capabilities. Therefore, this serves as a valuable addition to adverse event reporting by healthcare professionals during clinical trials focused on tumors (54).

HHIA-S, ETDQ-7 and PRO-CTCAE complement one another in evaluating hearing impairment from separate perspectives (Table IV): HHIA-S measures psychosocial hearing handicap in daily life, ETDQ-7 assesses eustachian tube-related conductive ear symptoms, and PRO-CTCAE provides standardized toxicity grading of treatment-related hearing loss for clinical trials. Concurrent administration of the three tools achieves full-spectrum auditory outcome assessment integrating middle ear symptoms, functional handicap and treatment adverse events.

## 5. Rehabilitative measures for hearing loss

### Eustachian tube insufflation

Eustachian tube insufflation is a treatment method that aims to loosen adhesions, reduce swelling and reopen the Eustachian tube passage in order to restore healthy ventilation functions through the introduction of air into the tube (Table V) (55-59). This technique eliminates negative pressure in the middle ear, thereby increasing the oxygen partial pressure within the middle ear, facilitates efficient mucus diffusion and accelerates fluid absorption and drainage. Moreover, Eustachian tube insufflation is a useful therapeutic option for secretory otitis media-related hearing loss and can be considered as part of the overall management of otitis media with effusion, alongside other approaches such as watchful waiting, hearing surveillance, periodic reevaluation, and surgical intervention when indicated, particularly tympanostomy tube insertion with or without adenoidectomy depending on the child's age (60). According to published auto-inflation protocols, the patient sits upright while a probe tip is inserted into one nostril and the other nostril is manually compressed. The patient holds a small amount of water in the mouth, airflow is delivered into the nasal cavity, and swallowing is performed after 1-2 sec of airflow to facilitate middle ear inflation. The procedure may be performed twice daily and should be suspended temporarily if an upper respiratory tract infection occurs (61,62).

### Inhalation of a high concentration of hydrogen-oxygen mixture

Inhalation of a high concentration of hydrogen-oxygen mixture (containing 2.0 l/min hydrogen and 1.0 l/min oxygen, equivalent to ~66.7% hydrogen and 33.3% oxygen by flow) may enhance the immune function of patients with low levels of immunity, such as individuals aged ≥65 years or those with chronic lung diseases or malignant tumors. This method aims to increase the quantity and activity of functional helper T lymphocytes, cytotoxic T lymphocytes and natural killer cells (63,64). Inhalation of small doses (typically 1-4%) of hydrogen gas may selectively reduce cytotoxic reactive oxygen species, particularly hydroxyl radicals, by reacting with them rather than by preventing their release. This antioxidant action is novel and effective, and it helps to protect hair cells in the auditory system and improve hearing (65). During this process, patients are administered continuous once-daily treatment for 4-12 weeks, with each session lasting 3-4 h. Patients are required to inhale a mixture of hydrogen-oxygen gas through a nasal cannula, at a rate of 2 liters/min hydrogen gas and 1 liter/min oxygen (~66.7% hydrogen and 33.3% oxygen by flow). The results of a previous study demonstrate that, after 4 weeks of hydrogen inhalation, the Eustachian Tube Dysfunction Questionnaire-7 (ETDQ-7) score, as well as air conduction and bone conduction thresholds, were markedly reduced, indicating alleviation of Eustachian tube dysfunction-related symptoms. Furthermore, in patients receiving radiotherapy or radiochemotherapy, air conduction thresholds were notably reduced. In total, 5 patients (10 ears) underwent hearing examinations 6-9 months after discontinuing hydrogen inhalation. Hearing remained stable or continued to improve in 3 of the 10 ears, although overall hearing thresholds increased during follow-up (Table V) (56).

### Acupuncture

Acupuncture, a distinct therapeutic modality of Traditional Chinese Medicine, is used for the management of diverse health conditions and symptoms, including improvements in maximum heart rate, pain, swelling, explosive force production and joint mobility. Acupuncture requires the insertion of sterile needles into specific areas of the body to regulate blood flow (66). Acupuncture may increase local blood circulation, optimize metabolism and facilitate blood flow in the ears through modulating inflammatory responses (67). A systematic review demonstrates that a combination of acupuncture with pharmacotherapy leads to improvements in the treatment of SSHL, compared with pharmacotherapy alone (68). Therefore, acupuncture may exhibit potential as an adjunctive treatment option for SSHL (69). To evaluate the role of acupuncture in reducing SSHL risk among patients with NPC, a nested case-control study was conducted. Among 811 patients with initial-onset SSHL, 27 individuals received acupuncture treatment (57). The results of the aforementioned study reveal that patients receiving acupuncture exhibit a reduced likelihood of developing SSHL, compared with the 784 individuals who did not receive such intervention [adjusted odds ratio, 0.39; 95% confidence interval, 0.25-0.60]. Therefore, incorporating acupuncture into routine care for patients with NPC resulted in a 61% decrease in their susceptibility to developing SSHL. Moreover, the observed effect is considered dose-dependent, as an increased frequency of acupuncture treatments corresponded to reduced incidence rates of SSHL (Table V) (57).

### Methylprednisolone use

A study by Chen *et al* (58) investigates the efficacy of methylprednisolone in treating radiation-induced hearing loss in patients with NPC undergoing radiotherapy. In the aforementioned study, a total of 25 patients receive a 14-day course of intravenous pulse therapy with methylprednisolone, in combination with radiotherapy. This group is compared with 28 patients who receive radiotherapy alone. Pure tone audiometry, distortion product otoacoustic emission (DPOAE) and auditory brainstem responses were evaluated prior to treatment, and 1 year after the completion of radiotherapy. The results of the aforementioned study reveal that the control group, who received radiotherapy alone, exhibit increased air-bone gap thresholds and decreased DPOAE levels at the 1-year follow-up assessment. By contrast, the treatment group demonstrate reduced pure tone air conduction thresholds and increased DPOAE levels, compared with the control group. Therefore, administration of methylprednisolone during radiotherapy may alleviate early SNHL caused by radiation exposure (Table V) (58).

In a further randomized study, 99 patients were administered either systemic infusion of methylprednisolone alone, or a combined treatment involving intratympanic dexamethasone and systemic methylprednisolone. The results of the aforementioned study indicate that both treatment groups achieve improvements in hearing over a 3-month follow-up period (Table V) (59).

## 6. Discussion

**Figure 1:**
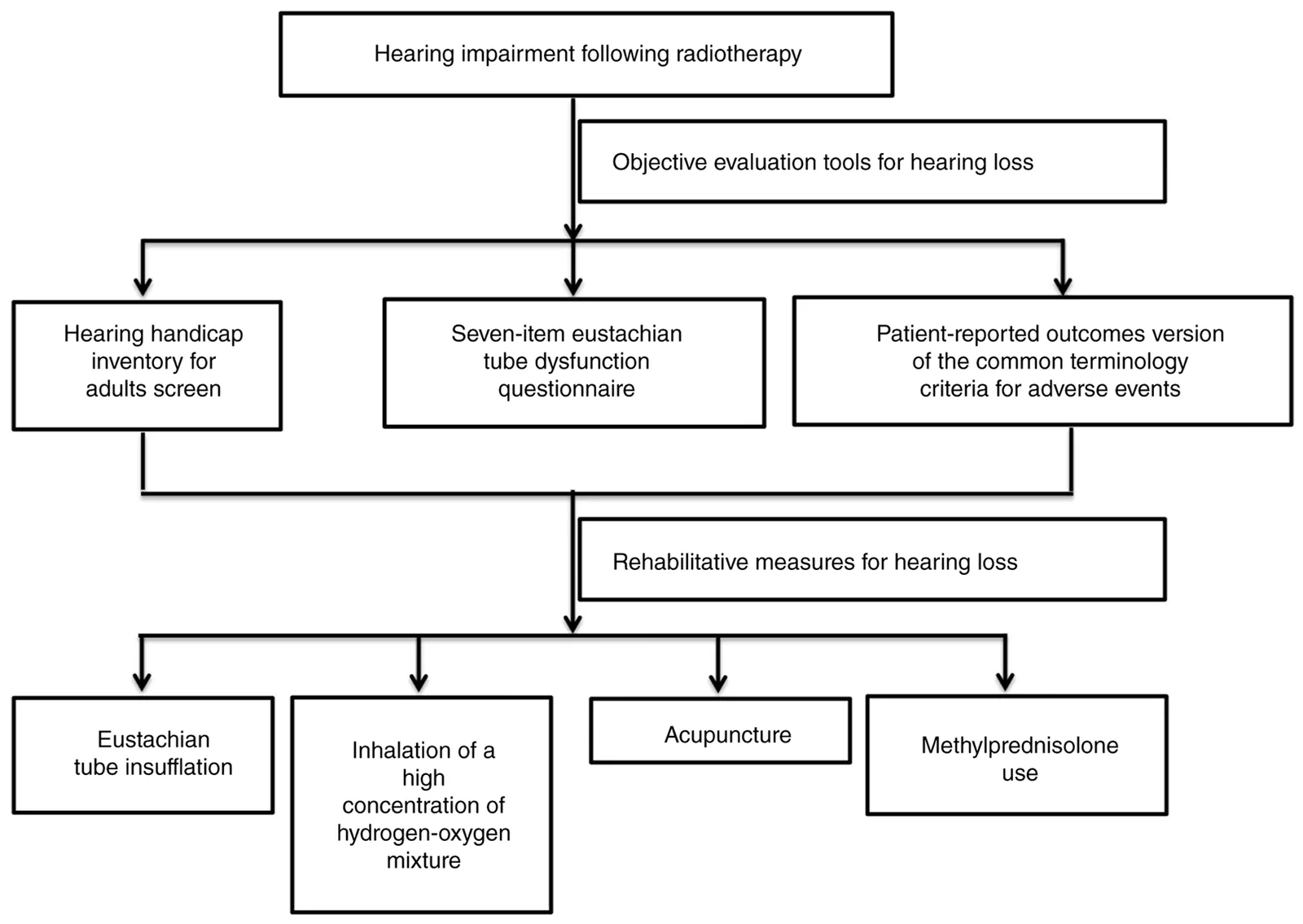
Concept map. Schematic overview of the assessment and rehabilitation pathway for hearing impairment following radiotherapy. The figure illustrates the objective evaluation tools used to assess hearing loss and the corresponding rehabilitative interventions.

The present review provides a comprehensive overview of the classification, assessment tools and intervention measures for hearing loss following radiotherapy for NPC, offering valuable insights for clinical practice (Fig. 1). Based on the underlying pathological mechanisms, hearing loss is categorized into three types, namely: CHL, SNHL and MHL. Each type of hearing loss exhibits distinct pathological and physiological processes as well as clinical manifestations. Hearing loss not only diminishes the quality of life of patients, but also exerts profound impacts on their cognitive function and emotional well-being. Hearing loss may increase the risk of cognitive impairments, including mild cognitive impairment, cognitive decline and dementia, and is associated with mental disorders, such as anxiety and depression. A study by Ho *et al* (26) investigates the association between the incidence of hearing loss and radiation dose-toxicity in patients with NPC. The results of the aforementioned study reveal numerous strategies that may mitigate the risk of SNHL in patients who have been previously treated for NPC. These strategies include constraining the radiation dose cochlear D-mean to <44-50 Gy, response-adaptive reduction of radiotherapy volume and dose, individualized cochlear constraints according to tumor stage, using specific radiotherapy planning techniques, and reducing systemic therapy. One such planning approach involves stratifying the anatomical structures adjacent to the nasopharynx into different layers while considering patterns of tumor spread. Target volumes are then delineated based on the extent of the primary tumor, using a combination of geometric expansion and inclusion of structures at risk of tumor involvement (26).

The present review aimed to summarize the characteristics of two other types of hearing loss, namely: CHL and MHL. The present study evaluated assessment tools, such as HHIA-S, ETDQ-7 and PRO-CTCAE. In addition, the present study aimed to investigate a range of treatment modalities, such as Eustachian tube insufflation, hydrogen-oxygen mixed gas inhalation therapy, acupuncture and methylprednisolone use, which may demonstrate therapeutic potential in preserving auditory function.

The multidisciplinary team (MDT) treatment model for radiation-induced hearing loss requires the integration of multidisciplinary resources and the development of individualized treatment plans, according to levels of damage and individual patient differences. Close collaboration between departments of otolaryngology, radiation oncology, radiology and rehabilitation is required. The department of otolaryngology, in conjunction with the department of imaging, should complete the classification of hearing damage and identify the cause, such as middle ear effusion or nerve damage. The department of radiation oncology should then review radiation records to analyze the spatiotemporal association between dose distribution and damage. Subsequently, the department of rehabilitation should guide the fitting of hearing aids and auditory rehabilitation training to improve the quality of life of the patient. Treatment of CHL involves medical intervention, such as ototoxic ear drops. If ineffective, tympanocentesis or tube placement may be used. The early use of steroid pulse therapy combined with neurotrophic drugs, such as mecobalamin, is recommended for SHL, and hearing aids should be fitted for patients with moderate to severe levels of the condition. Regular MDT assessments should be conducted according to a risk-adapted schedule, typically every 3-6 months during the first 3 years after treatment, every 6-12 months during years 4-5 and annually thereafter, to monitor treatment outcomes and complications and to allow timely adjustment of the management plan.

Further studies are required to investigate more effective and safe intervention measures for the treatment of NPC. Emphasis should be placed on interdisciplinary integration and comprehensive research on hearing protection. Moreover, with advancements in artificial intelligence and the analysis of large datasets, future developments should focus on creating personalized systems to monitor the hearing status of patients in real-time, which may lead to precise treatment interventions. Additionally, enhancing healthcare professional-patient communication and educating patients on the importance of hearing protection are important for improving post-radiotherapy quality of life for patients with NPC. In conclusion, addressing the complexities of hearing loss following radiotherapy for NPC necessitates collaborative efforts from both the medical community and society as a whole, to promote further research development, and provide comprehensive and effective treatment options.

## Figures and Tables

**Table I:** Hearing handicap inventory for adults screen (47).

Item	Question^a^	Yes (4)	Sometimes (2)	No (0)
S-1.	Does a hearing problem cause you to use the phone less often than you would like?			
E-2.^b^	Does a hearing problem cause you to feel embarrassed when meeting new people?			
S-3.	Does a hearing problem cause you to avoid groups of people?			
E-4.	Does a hearing problem make you irritable?			
E-5.^b^	Does a hearing problem cause you to feel frustrated when talking to members of your family?			
S-6.	Does a hearing problem cause you difficulty when attending a party?			
S-7.^b^	Does a hearing problem cause you difficulty hearing/understanding coworkers, clients, or customers?			
E-8.^b^	Do you feel handicapped by a hearing problem?			
S-9.^b^	Does a hearing problem cause you difficulty when visiting friends, relatives, or neighbors?			
E-10.	Does a hearing problem cause you to feel frustrated when talking to coworkers, clients, or customers?			
S-11.^b^	Does a hearing problem cause you difficulty in the movies or theater?			
E-12.	Does a hearing problem cause you to be nervous?			
S-13.	Does a hearing problem cause you to visit friends, relatives, or neighbors less often than you would like?			
E-14.^b^	Does a hearing problem cause you to have arguments with family members?			
S-15.^b^	Does a hearing problem cause you difficulty when listening to TV or radio?			
S-16.	Does a hearing problem cause you to go shopping less often than you would like?			
E-17.	Does any problem or difficulty with your hearing upset you at all?			
E-18.	Does a hearing problem cause you to want to be by yourself?			
S-19.	Does a hearing problem cause you to talk to family members less often than you would like?			
E-20.^b^	Do you feel that any difficulty with your hearing limits or hampers your personal or social life?			
S-21.^b^	Does a hearing problem cause you difficulty when in a restaurant with relatives or friends?			
E-22.	Does a hearing problem cause you to feel depressed?			
S-23.	Does a hearing problem cause you to listen to TV or radio less often than you would like?			
E-24.	Does a hearing problem cause you to feel uncomfortable when talking to friends?			
E-25.	Does a hearing problem cause you to feel left out when you are with a group of people?			

^a^Instructions: The purpose of the scale is to identify the problems your hearing loss may be causing you. Check Yes, Sometimes, or No for each question. Do not skip a question if you avoid a situation because of a hearing problem.

^b^Items comprising the HHIA-S.

**Table II:** Seven-item Eustachian tube dysfunction questionnaire (50).

Over the past 1 month, how much has each of the following been a problem for you?	No problem			Moderate problem			Severe problem
1. Pressure in the ears?	1	2	3	4	5	6	7
2. Pain in the ears?	1	2	3	4	5	6	7
3. A feeling that your ears are clogged or ‘under water’?	1	2	3	4	5	6	7
4. Ear symptoms when you have a cold or sinusitis?	1	2	3	4	5	6	7
5. Crackling or popping sounds in the ears?	1	2	3	4	5	6	7
6. Ringing in the ears?	1	2	3	4	5	6	7
7. A feeling that your hearing is muffled?	1	2	3	4	5	6	7

**Table III:** Patient-reported outcomes version of the common terminology criteria for adverse events (53).

CTCAE Term	Grade 1	Grade 2	Grade 3	Grade 4	Grade 5
Ear pain	Mild pain	Moderate pain; limiting instrumental ADL	Severe pain; limiting self care ADL	-	-
Definition	A disorder characterized by a sensation of marked discomfort in the external ear region				
Hearing impaired	Adults enrolled on a Monitoring Program (on a 1, 2, 4, 3, 6, and 8 kHz audiogram): Threshold shift of 15-25 dB averaged at 2 contiguous test frequencies in at least one ear; Adults not enrolled on a Monitoring Program: Subjective change in hearing in the absence of documented hearing loss; Pediatric (on a 1, 2, 3, 4, 6, and 8 kHz audiogram): Threshold shift >20 dB hearing loss (HL) (i.e., 25 dB HL or greater); sensorineural hearing loss (SNHL) above4 kHz (i.e., 6 or 8 kHz) in at least one ear	Adults enrolled on a Monitoring Program (on a 1, 2, 3, 4, 6, and 8 kHz audiogram): Threshold shift of >25 dB averaged at 2 contiguous test frequencies in at least one ear; Adults not enrolled on a Monitoring Program: Hearing loss with hearing aid or intervention not indicated; limiting instrumental ADL; Pediatric (on a 1, 2, 3, 4, 6, and 8 kHz audiogram): Threshold shift >20 dBat 4 kHz in at least one ear	Adults enrolled on a Monitoring Program (on a 1, 2, 3, 4, 6, and 8 kHz audiogram): Threshold shift of >25 dB averaged at 3 contiguous test frequencies in at least one ear; therapeutic intervention indicated; Adults not enrolled on a Monitoring Program: Hearing loss with hearing aid or intervention indicated; limiting self care ADL; Pediatric (on a 1, 2, 3, 4, 6, and 8 kHz audiogram): Hearing loss sufficient to indicate therapeutic intervention, including hearing aids; threshold shift >20 dB at 2 to <4 kHz in at least one ear	Adults: Decrease in hearing to profound bilateral loss (absolute threshold >80 dB HL at 2 kHz and above); nonserviceable hearing Pediatric:Audiologic indication for cochlear implant; >40 dB HL (i.e., 45 dB HL or more); SNHL at 2 kHz and above	-
Definition:	A disorder characterized by partial or complete loss of the ability to detect or understand sounds resulting from damage to ear structures.				

CTCAE, common terminology criteria for adverse events.

**Table IV:** Comparison of hearing impairment assessment tools.

Tool	HHIA-S	ETDQ-7	PRO-CTCAE
Application	Preliminary screening for hearing impairment	Evaluation of symptoms associated with Eustachian tube dysfunction	Assessment of adverse events in oncology clinical trials
Items	A total of 10 items (five emotional and five social/situational)	A total of seven questions	Domains: Frequency, severity and impact of adverse events
Scoring	Scored zero points for ‘No’, two points for ‘Sometimes’ and four points for ‘Yes’	Scored one to seven points (based on symptom severity)	A one to five graded scale
Score cut-off	Impairment indicated by a score of ≥8 (8-24, mild; >24, severe)	A score of ≥14.5 indicates Eustachian tube dysfunction	Grade 5, mortality; grade 4, life-threatening toxicity requiring intervention
Localization	Chinese version validated, no modification was made to the questionnaire's official name	Chinese version validated, no modification was made to the questionnaire's official name	Chinese version validated, no modification was made to the questionnaire's official name
Country	USA	USA	USA
Strengths	Self-administered, simple operation, and no specialized equipment required	Easy symptom collection and high operability	Broad applicability and complements clinician-reported adverse event data
Limitations	Subjective bias and requires objective screening for specific hearing loss	Fails to differentiate obstructive Eustachian tube dysfunction vs. patulous Eustachian tube dysfunction	None
Population	Adults (28-59 years)	Mean age, 49.8±13.9 in the Eustachian tube dysfunction group and 53.4±11.2 years in the control group	Median age, 59 years
Reliability	Original test: Test-retest reliability, *r*=0.930. Chinese test: Sensitivity, 68.4%; and specificity, 96.7%	Original test: Cronbach's, α=0.711; and test-retest reliability, *r*=0.780. Chinese test: Cronbach's, α=0.897; sensitivity, 96.1%; and specificity, 85.0%	Original test: Internal consistency, α=0.760; and test-retest reliability, *r*=0.700-0.910. Chinese test: Cronbach's, α=0.900

HHIA-S, hearing handicap inventory for adults screen; USA, United States of America; ETDQ-7, seven-item Eustachian tube dysfunction questionnaire; PRO-CTCAE, patient-reported outcomes version of the common terminology criteria for adverse events.

**Table V:** Summary of the therapeutic approaches and studies on hearing loss associated with nasopharyngeal carcinoma.

First author, year	Therapeutic approach	Mechanism of action	Specific procedures	Study design and sample size	Statistical data	Qualitative data	(Refs.)
Fermo *et al*, 2022	Eustachian tube inflation therapy.	Relieves adhesions, restores ventilation function of the Eustachian tube, eliminates negative pressure in the middle ear and promotes effusion absorption and drainage.	Patients hold water in their mouth, place the silicone treatment head against one nostril, seal both nostrils and inhale air for ~3 sec. Subsequently, patients swallow the water while the device is running. Repeat twice daily for 6-8 weeks.	None.	None.	In total, 50 patients were cured^a^, 3 patients improved^b^ and 1 patient was unaffected^c^.	(55)
Kong *et al*, 2022	Hydrogen-oxygen inhalation. therapy	Enhances immune function, antioxidant effects, protects auditory hair cells and improves hearing.	Patients inhale a mixture of hydrogen (2 l/min) and oxygen (1 l/min) via a nasal cannula for 3-6 h daily, for 4-12 weeks.	Prospective cohort study, the sample size was formed of 17 patients. Follow-up duration, the median time from radiotherapy to present was 228 months. The median time from hearing loss diagnosis to present was 92 months.	Notable reduction in Eustachian tube dysfunction score, air conduction threshold and bone conduction threshold after 4 weeks of hydrogen inhalation. The pre-inhalation air conduction threshold was 74.69±27.03 dB. At week 12, the lowest air conduction threshold was 66.88±20.88 dB. Pre-inhalation bone conduction threshold was 45.70±21.58 dB. At week 12, the lowest bone conduction threshold was 40.94±18.93 dB.	In a real-world evidence investigation examining voluntary hydrogen inhalation as a rehabilitation method among patients with cancer, a 63-year-old male with nasopharyngeal carcinoma experienced hearing loss post-radiotherapy and relied on a hearing aid. Starting daily hydrogen inhalation in January 2019, the patient achieved notable hearing improvement after 3 months and no longer required the hearing aid.	(56)
Shen *et al*, 2024	Acupuncture. therapy	Regulates inflammatory responses, improves local blood circulation and promotes blood flow in the ears.	Acupuncture was performed by inserting sterile needles into predefined anatomical sites. Patients diagnosed with NPC who received at least six acupuncture treatments were identified as ‘acupuncture users’, while the remaining cases were considered ‘non-acupuncture users’.	Cohort study formed of 811 patients with SSHL and 1,452 patients with NPC without SSHL. It had a 10-year study period.	Acupuncture notably reduced the risk of SSHL in patients with NPC, with an adjusted OR of 0.39 (95% CI, 0.25-0.60). The longer the duration of acupuncture use, the greater the reduction in SSHL risk in a dose-dependent manner.	None.	(57)
Chen *et al*, 2016s	Methylpredni-solone intravenous pulse therapy	Mitigates early sensorineural hearing loss caused by radiotherapy and protects outer hair cells.	Methylprednisolone intravenous pulse therapy for 14 days compared with radiotherapy alone.	Prospective, controlled clinical study. Treatment group was formed of 25 patients, and the control group was formed of 28 patients. The follow-up duration was 1 year.	Following 1 year after radiotherapy, the control group showed increased air and bone conduction pure tone hearing thresholds and decreased DPOAE levels. No differences were observed in ABR wave I, III and V latencies and I-V interwave latencies before and after radiotherapy. The treatment group had decreased air conduction thresholds and increased DPOAE levels compared with the control group.	None.	(58)
Lyu *et al*, 2021	Intratympanic methylpredni-solone injection	Downregulates inflammatory responses, promotes cochlear blood flow, inhibits inner ear cell apoptosis, improves stria vascularis function and maintains ionic homeostasis.	Group one: Treatment was initiated on day 1 with intravenous dexamethasone (10 mg/day for 5 days, followed by 5 mg/day for 7 days) and intratympanic methylprednisolone (40 mg), which was administered every other day for a total of four doses. Group two: Intravenous dexamethasone (10 mg/day for 5 days, followed by 5 mg/dayfor 7 days) and intratympanic methylprednisolone (40 mg) every other day for 4 times starting on day 6.	Retrospective study with a total number of 61 patients. The follow-up duration was 6 months.	Both groups showed comparable PTA gains with notable improvement compared with pre-treatment. The long-term hearing threshold data (follow-up at 6 months) showed no notable changes between 3 months and 6 months post-treatment for both groups (at 6 months: Group one, 40.50±21.76 dB and group two, 38.79±20.04 dB; and at 3 months: Group one, 39.27±22.28 dB and group two, 37.61±20.57 dB), indicating that patients achieved maximum benefit from short-term treatment.	In group one, 5 patients (16.67%) were classified as ineffective, 13 patients (43.33%) as effective and 12 patients (40.00%) as notably effective. In group two, 2 patients (6.45%) were classified as ineffective, 10 patients (32.26%) as effective and 19 patients (61.29%) as notably effective.	(59)

^a^Symptoms completely resolved, hearing restored to normal levels, tympanogram shows type A, stapedial reflex is elicitable and the tympanic membrane appears normal.

^b^Symptoms alleviated, hearing partially restored but not to normal levels, tympanogram transitions from type B to C or from type C to A, with mild tympanic membrane retraction or minimal effusion.

^c^No improvement in symptoms, type B tympanogram, notable tympanic membrane retraction and persistent middle ear effusion. DPOAE, distortion product otoacoustic emission; ABR, auditory brainstem responses; NPC, nasopharyngeal carcinoma; SSHL, sudden sensorineural hearing loss; PTA, pure tone average; dB, decibel; OR, odds ratio; CI, confidence interval.

## Data Availability

Not applicable.
